# Intravenous immunoglobulin-induced eczematous dermatitis treated with dupilumab

**DOI:** 10.1016/j.jdcr.2024.05.002

**Published:** 2024-05-10

**Authors:** Katelyn Singh, Rachel Breidbart, Anjali Jaiswal, William Damsky, Keith A. Choate, Matthew Vesely

**Affiliations:** a Department of Dermatology, Yale School of Medicine, New Haven, Connecticut; b Department of Pathology, Yale School of Medicine, New Haven, Connecticut; c Department of Genetics, Yale School of Medicine, New Haven, Connecticut

**Keywords:** dermatitis, drug reactions, dupilumab, intravenous immunoglobulin

## Introduction

Intravenous immunoglobulin (IVIG) therapy is an effective treatment for immunodeficiency and autoimmune diseases. IVIG is well tolerated, with the most common adverse reaction being a transient flu-like illness. Rarely, severe adverse effects such as renal impairment and aseptic meningitis can occur. Adverse dermatological reactions are uncommon, with a reported incidence of 6%, which include urticaria, maculopapular rash, and desquamation.^1^ Rare cases of IVIG-associated eczematous reactions have also been reported in the literature.^1^, ^2^, ^3^, ^4^, ^5^, ^6^, ^7^ Here, we report 2 cases of eczematous eruptions that developed shortly after IVIG infusions and were successfully treated with dupilumab.

## Case report

### Case 1

A 78-year-old man with a history of non-Hodgkin B-cell lymphoma and progressive muscle weakness was referred for evaluation of a new pruritic rash. No previous history of eczema, allergic rhinitis, or asthma was documented. A few days prior to rash onset, the patient received an IVIG infusion (0.5 g/kg/d for 4 days) for suspected Lambert-Eaton myasthenic syndrome. The patient was initiated on oral prednisone 40 mg daily for 7 days and the rash resolved.

**Fig 1:**
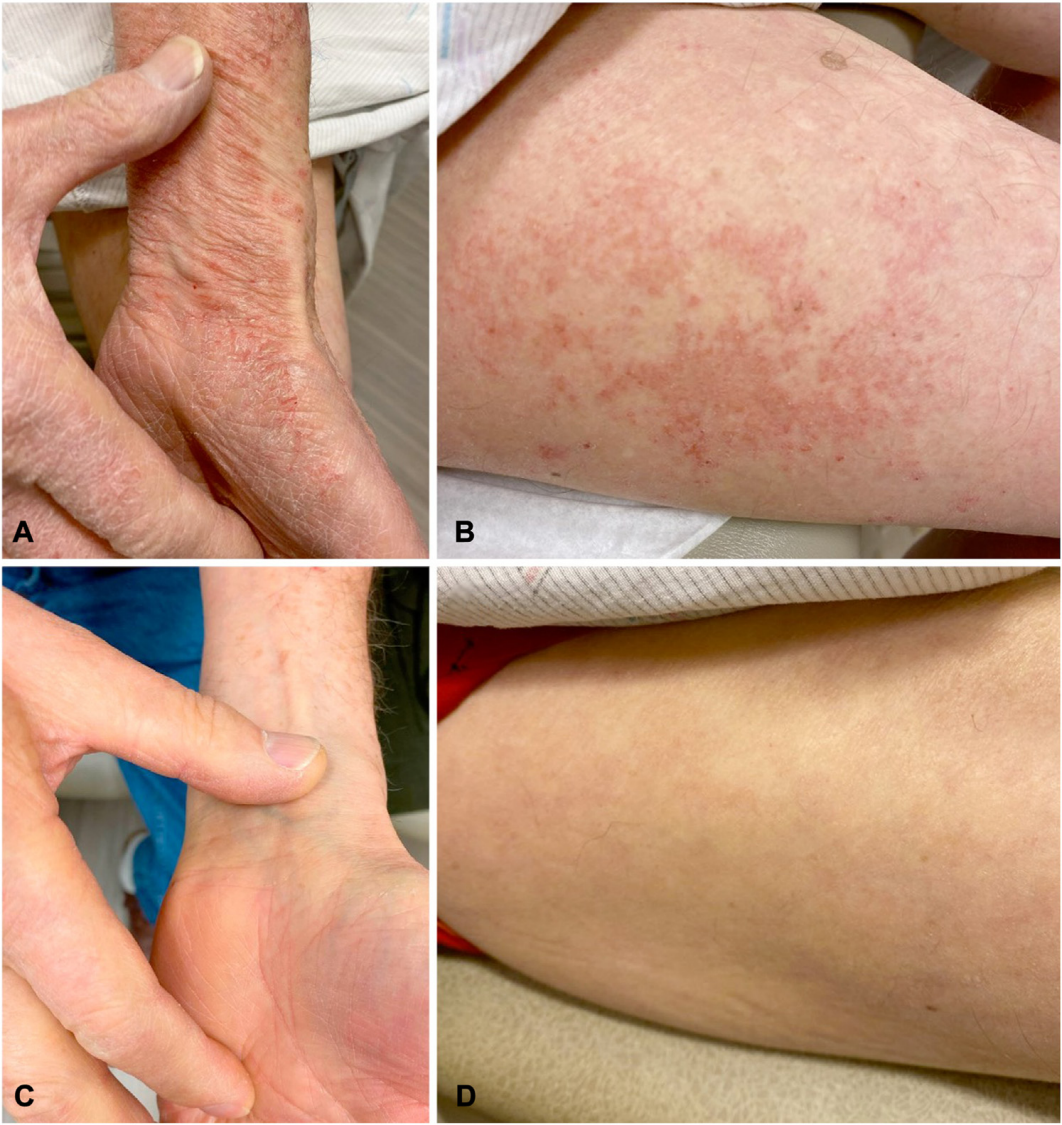
Clinical images of patient 1. Appearance of hands (**A**) and right outer thigh (**B**) before and after (**C** and **D**) dupilumab therapy 300 mg every 2 weeks for 3 months for the treatment of IVIG-induced eczematous dermatitis.

**Fig 2:**
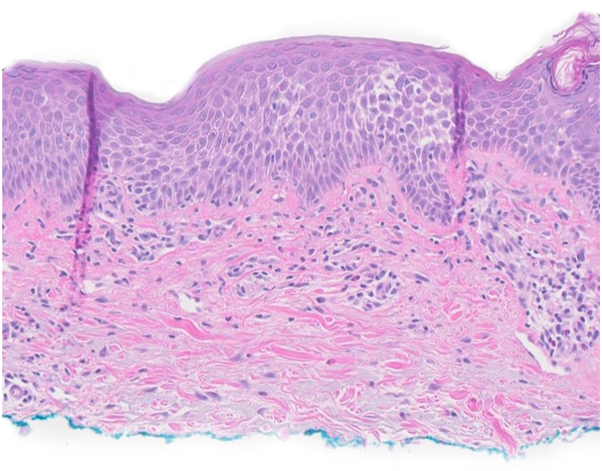
Histopathology of biopsy. Histological evaluation of skin biopsy from right volar wrist shows spongiosis, lymphocytic infiltrate, and a few scattered eosinophils consistent with an eczematous process (hematoxylin and eosin, original magnification 40×).

The rash reoccurred approximately 1 week after a subsequent dose of IVIG. Physical examination revealed confluent pink, scaly, lichenified plaques involving the trunk, extremities, face, and hands with islands of sparing involving greater than 80% body surface area (Fig 1, *A* and *B*). Polymyositis and dermatomyositis panels were negative. Shave biopsies from 2 sites revealed spongiotic dermatitis compatible with an eczematous process (Fig 2). Findings were not suggestive of psoriasis, fungi, connective tissue disease, or cutaneous T-cell lymphoma. Triamcinolone 0.1% cream was initiated, followed by topical clobetasol 0.05% ointment and short courses of oral prednisone of 40 mg daily for 7 to 14 days, but the patient continued to have persistent severe eczema involving 20% body surface area with frequent flares. Dupilumab 300 mg subcutaneous injections were started after an initial loading dose of 600 mg. After 2 months of treatment with dupilumab, there was significant improvement, with only pink eczematous plaques remaining on the bilateral lower legs, equaling a 5% body surface area (Fig 1, *C* and *D*). After 6 months of treatment with dupilumab in the setting of continued IVIG therapy, he had total resolution of his eczema.

### Case 2

**Fig 3:**
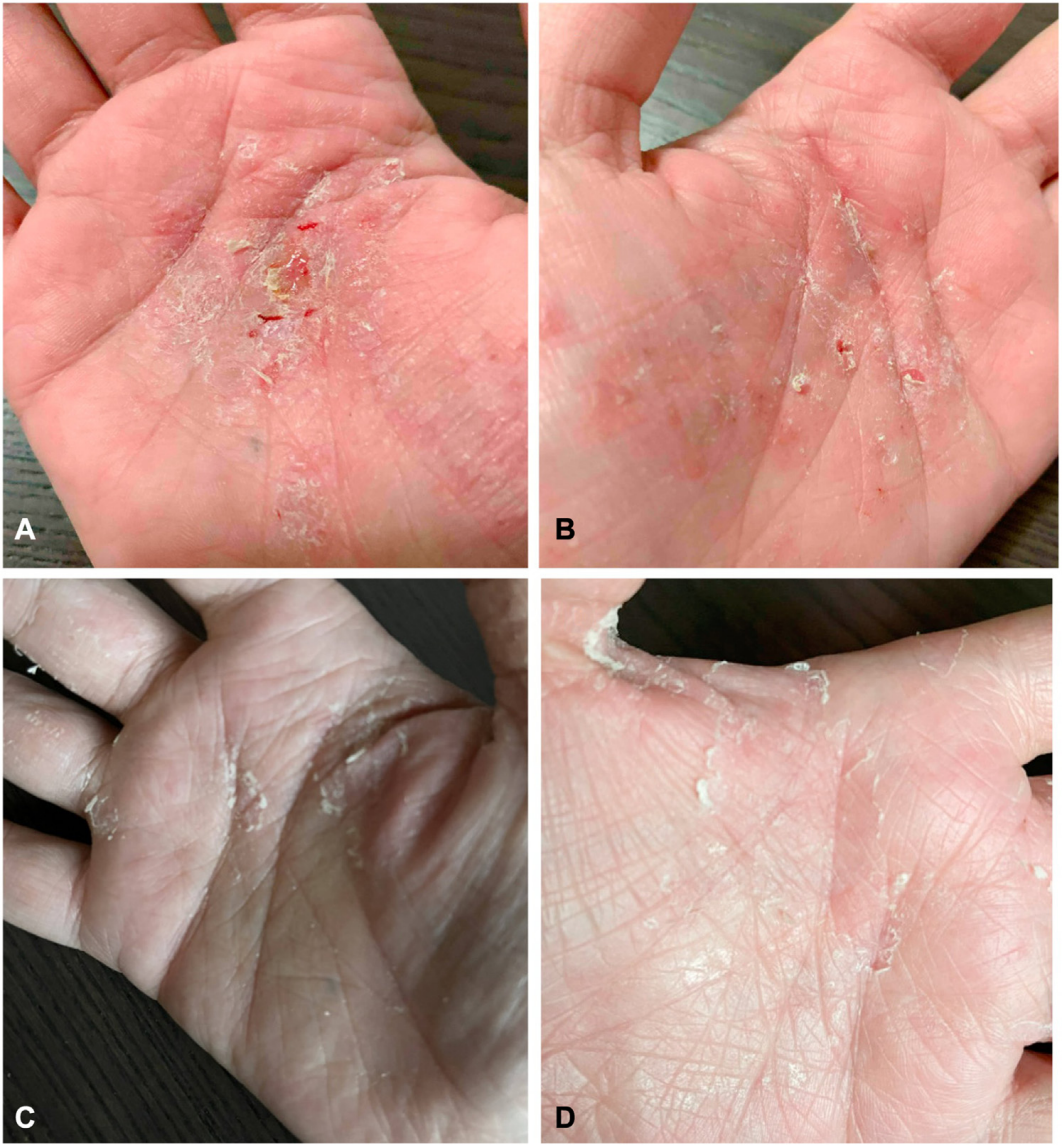
Clinical images of patient 2. Appearance of right palm (**A**) and left palm (**B**) prior to treatment with dupilumab show thick eczematous plaques with scale and fissuring. Appearance of right palm (**C**) and left palm (**D**) 7 months after treatment with dupilumab show significant improvement.

A 40-year-old female with a history of celiac disease and chronic inflammatory demyelinating polyneuropathy was referred for an eczematous reaction in the setting of IVIG treatment. Prior to rash onset, the patient developed diffuse paresthesias and weakness, leading to a diagnosis of chronic inflammatory demyelinating polyneuropathy. The patient was initiated on IVIG 2 g/kg every 3 weeks. IVIG treatments were complicated by the development of a pruritic rash that began on the palms and progressively worsened with each subsequent infusion, eventually spreading to the rest of the body. On physical examination, bilateral palms were significant for thick eczematous plaques with scale and fissuring (Fig 3, *A* and *B*). Legs were notable for oozing eczematous plaques with erosions and excoriations. Laboratory work-up was negative for bullous pemphigoid antibodies and presentation was consistent with IVIG-induced eczematous reaction.

The patient was started on prednisone 40 mg once daily for 7 days with taper and experienced initial improvement in eczematous symptoms. Triamcinolone 0.1% cream, clobetasol 0.05% ointment, crisaborole 2% ointment, and tacrolimus 0.1% ointment failed to lead to notable improvement. Given extent of disease, and failure of systemic and topical steroids in addition to steroid-sparing agents such as crisaborole 2% ointment and tacrolimus 0.1% ointment, dupilumab was initiated. Dupilumab 300 mg subcutaneous injections were started after an initial loading dose of 600 mg resulting in near resolution of eczema with no flares over the next 3 months (Fig 3, *C* and *D*). Additionally, there was an attempt to transition IVIG to rituximab; however, the patient experienced clinical worsening requiring restarting IVIG 2 g/kg with spacing increased to every 4 weeks with the addition of methylprednisolone 250-mg infusion on the first day of IVIG infusion. Dupilumab was continued throughout, allowing the patient to tolerate IVIG treatments without eczema recurrence.

## Discussion

Evidence of eczematous reactions as an adverse reaction of IVIG therapy has been increasingly recognized as the Food and Drug Administration-approved indications and off-label uses for IVIG continue to expand. We reported eczematous reactions soon after the initial IVIG infusion that reoccurred following subsequent IVIG administration with greater severity, which is consistent with reported cases of IVIG-induced eczema.^2^^,^^5^ The most commonly described eczematous adverse reaction is a localized pompholyx reaction on the palms.^1^^,^^3^ However, in this report, we described a case of a widespread eczematous eruption without pompholyx (patient 1), which has been observed in less than 5% of patients with IVIG-induced eczema.^4^ In addition, the adverse eczematous reactions were not associated with a prior history of atopic disorders, which is consistent with the literature.^3^

IVIG-induced eczema is commonly treated with topical steroids; however, severe cases often require oral steroids and lead to IVIG discontinuation.^1^^,^^3^^,^^5^^,^^6^ Our report shows that dupilumab is effective in treating widespread IVIG-induced eczema that was refractory to other therapies, allowing IVIG treatment to be continued without the risks associated with frequent high-dose systemic corticosteroids.

In summary, this case provides further evidence that eczematous reactions can be induced by IVIG therapy. It also shows that dupilumab can be an effective treatment option. As IVIG use becomes more prevalent in treating various conditions, so will the incidence of eczematous reactions as an adverse effect of IVIG, requiring increased awareness of effective therapeutic options.

## Conflicts of interest

Dr Vesely’s spouse is an employee of Regeneron Pharmaceuticals, the maker of dupilumab. Dr Damsky is a consultant for Pfizer, Eli Lilly, and TWi Biotechnology, and receives licensing fees from EMD/Millipore/Sigma. Dr Singh, Authors Breidbart, Jaiswal, and Dr Choate have no conflicts of interest to declare.
